# Gut Microbiota Alterations from Three-Strain Yogurt Formulation Treatments in Slow-Transit Constipation

**DOI:** 10.1155/2020/4583973

**Published:** 2020-02-18

**Authors:** Xiao-Ran Li, Chen-Jian Liu, Xiao-Dan Tang, He-Ming Zhang, Yi-Yong Luo, Le Zhang, En yang

**Affiliations:** ^1^Faculty of Life Science and Technology, Kunming University of Science and Technology, Chenggong, Kunming 650500, Yunnan, China; ^2^Department of Gastroenterology, First People Hospital of Yunnan Province, Kunming 650032, Yunnan, China; ^3^Department of Gastroenterology, The Affiliated Hospital of Kunming University of Science and Technology, Kunming 650032, Yunnan, China; ^4^Institute for Integrative Genome Biology, University of California Riverside, Riverside, CA 92521, USA

## Abstract

The objective of this study was to evaluate the effects of a three-strain yogurt formulation in slow-transit constipation (STC) patients. Each individual in both treatment groups consumed 250 mL of the formulated yogurt daily for a week (7 days), and fecal samples were collected for gut microbiota and short-chain fatty acid (SCFA) analyses. A significant increase in the defection frequency (*p* < 0.001) and bacterial diversity (*p*=0.027) at the 100% sequence homology level and a decrease in the concentrations of acetic acid (*p*=0.014), propionic acid (*p*=0.019), and butanoic acid (*p*=0.005) were observed after the STC patients consumed three-strain yogurt formulation. In addition, the consumption of the three-strain yogurt formulation significantly altered the composition of the intestinal bacteria in the STC patients. The relative abundances of 23 genera in the top dominating genera were altered significantly after the STC patients consumed the yogurt. In summary, the consumption of 250 mL day^−^ the three-strain yogurt formulation described in this study can play a role in improving the symptoms of STC.

## 1. Introduction

Constipation is a common functional gastrointestinal (GI) disorder with clinical symptoms that include difficulty or infrequency in passing feces, prolonged GI emptying times, and small, dry, and hard feces [1]. The incidence of chronic constipation in Asian populations is 2.6 to 24.8% and is especially prevalent in women and the elderly [2]. Constipation can be divided into three broad categories: normal-transit constipation, slow-transit constipation (STC), and disorders of defecation or rectal evacuation (obstructive defecation) [3]. STC is considered to be a majority category, resulting in a decreased rate of colonic transit and accounting for 30% of total the incidence of constipation [4].

Constipation affects quality of life, and approximately a fifth of constipation patients had comorbid hypertension [5]. Osmotic or secretory laxatives, fecal softeners, and prokinetic agents are used to treat constipation. However, most of these therapies are often ineffective and may even lead to a cycle of drug abuse [6]. Thus, alternative, safe, and effective therapeutic options are still needed, such as probiotics. Probiotics are defined as live microorganisms that can play a beneficial role in the host when consumed in high enough amounts, including three well-known genera of microbes, *Lactobacillus*, *Bifidobacterium*, and *Saccharomyces* [7]. Probiotics can alter the colonic flora and may improve bowel functions, including with respect to the prevention and treatment of gastrointestinal disorders and inflammation associated with bowel disease [8]. There has been growing evidence suggesting that probiotics, or at least specific probiotic strains, may have a beneficial role in alleviating constipation symptoms [9, 10]. Interestingly, functional foods, such as probiotic-containing dairy products, have been introduced as treatment options for constipation and showed favorable effects [11–15].

Lactic acid bacteria (LAB) are widely used as probiotics in the fermentation industry due to their ability to improve food texture, alter flavor components, and develop the nutritional quality and health aspects of foods. In a previous study, three species of LAB, *Lactobacillus plantarum* 4_3, *Lactobacillus casei* AS02, and *Lactococcus lactis* BZ06, were used to prepare a yogurt formulation, where the *L. plantarum* strain was isolated from Chinese traditional fermented soybean and could produce folate, while the *L. casei* and *La. lactis* strains were isolated from a Yunnan traditional formulated dairy cake. In our previous study, we observed that the consumption of this novel three-strain yogurt formulation could increase the intestinal charcoal transit ratio and alter the intestinal bacterial community composition in loperamide-induced constipated mice [15]. Therefore, the two aims of this study were as follows: (1) to determine whether the three-strain yogurt formulation works in patients with STC and (2) to evaluate the bacterial community composition in the feces of these patients before and after consumption of the three-strain yogurt formulation.

## 2. Materials and Methods

### 2.1. Study Population and Experimental Design

Four healthy individuals with normal bowel habits and without any recent gastrointestinal symptoms were compared with sixteen patients who fulfilled the Rome III criteria and were selected from a cohort undergoing routine colon transit study assessment for STC at The First People's Hospital of Yunnan Province, Kunming, Yunnan Province, China. The study was approved by the institutional review board (CWO) of the Medical School of Kunming University of Science and Technology, Yunnan Province, China. All patients provided written informed consent. After inquiry, we observed that none of the patients had other intestinal diseases and that STC was caused by a lack of intestinal motility due to eating habits. Additional information on the characteristics of the study population is detailed in Table 1.

The comparative effects of the formulation yogurt [15] between healthy individuals and STC patients were studied. Both healthy individuals and the STC patients were asked to eat 250 mL of the three-strain yogurt formulation on an empty stomach every morning for 7 days. In addition, fecal samples were obtained before and after the 7-day treatment, and the collected samples were stored at −80°C, resulting in 40 samples being collected from 20 individuals. All fecal samples were divided into four groups: healthy individuals before yogurt consumption (HB), healthy individuals after yogurt consumption (HY), patients before yogurt consumption (PB), and patients after yogurt consumption (PY). More information on the study design is detailed in Table 1.

### 2.2. Short-Chain Fatty Acid (SCFA) Quantification

The short-chain fatty acids (SCFAs) in fecal samples were quantified within one month after collection. The fecal samples (0.5 g) were homogenized in 1 mL of methanol for 10 min, after which the fecal suspension was shaken, and the pH of the solution was adjusted to 2–3 using sulfuric acid. Subsequently, the suspension was ultrasonicated for 30 min and then centrifuged at 5,000 r min^−1^ for 15 min. Then, the supernatant was filtered into a gas bottle for GC analysis, and contents of SCFAs (acetic, propionic and butanoic acid) were calculated according to standard curves. The analysis of SCFAs was conducted using a gas chromatography system (GC7890, Agilent, USA) coupled to a flame ionization detector (FID) as previously described [15] with minor modifications. Briefly, the injection-port temperature was set to 200°C with a split ratio of 20 : 1. An Agilent 122–7032 DB-WAX column (30 m × 250 *μ*m, 0.25 *μ*m) was used with a helium flow of 1.2 mL min^−1^ and a sample volume of 2 *μ*L. The column temperature was initially set at 120°C for 0.5 min and then raised from 120 to 180°C at a rate of 8°C per min and maintained at 200°C for 5 min. Subsequently, the temperature was further increased to 240°C at a rate of 30°C per min, and the final temperature was held for 2 min. The FID detector temperature was held at 250°C.

### 2.3. Fecal Bacterial Analysis

Fecal samples were stored at −80°C prior to DNA extraction. Bacterial DNA was obtained from fecal samples using a QIAamp DNA Stool Mini kit (Qiagen, Germany) according to the manufacturer's instructions. Bacterial 16S rRNA gene amplification, heteroduplex elimination and sequencing procedures were conducted as previously described [16] with the following barcoded primers: 343F (5′-GAC AGT ACG GRA GGC AGC AG-3′) and 798R (5′-GAC AGA GGG TAT CTA ATC CT-3′). The PCR products were analyzed by gel electrophoresis with 1% (wt/vol) agars purified with a QIAquick Gel Extraction kit (QIAGEN, Germany) and quantified with a NanoDrop 3300 instrument (Thermo Fisher, USA). To obtain similar numbers of sequences from each sample, an equivalent amount of each purified PCR product was mixed for sequencing using an Illumina MiSeq™ instrument (Illumina, USA).

Sequences were removed from the analysis if they were <350 or >450 nt, contained ambiguous characters, contained an uncorrectable barcode, or did not contain the primer sequence. All sequences were extracted using the barcodes for each sample. Sequence processing was performed using Mothur v1.39.1 [17] according to the MiSeq SOP. The SSU rRNA database sequences and taxonomic information from SILVA (v128) were downloaded directly from the Mothur website. Sequences were assigned to operational taxonomic units (OTUs) performing alignments. To determine the phylogenetic position of the 16S rRNA genes, the top 25 most abundant OTUs were compared with available database sequences via BLAST searches, and related sequences were obtained from GenBank.

### 2.4. Statistics Analysis

Statistical analyses were performed using Statistica software v. 10.0 (StatSoft Inc., Tulsa, OK, USA). All results are presented as the means ± s.d. In all study populations, differences between data collected before and after ingestion of the yogurt formulation were investigated using one-way analysis of variance (ANOVA). *p* values <0.05 were considered statistically significant.

### 2.5. Nucleotide Sequences

The Illumina sequence data produced in this study were submitted in the Short Read Archive of NCBI under the accession number PRJNA510483.

## 3. Results and Discussion

### 3.1. Clinical Response

Table 1 summarizes the clinical responses of the STC patients after they consumed the three-strain yogurt formulation for 7 days. An increase in the frequency of defecation (*p* < 0.001) was observed in the STC patients during the 7 days the patients consumed the yogurt formulation. Similar results were obtained in our previous study, where the shortest time to the first defecation and a significant (*p* < 0.05) increase in the intestinal charcoal transit ratio was observed in the groups of mice receiving the three-strain yogurt formulation compared to the control and nonyogurt groups [15]. Similarly, patient consumption of 300 g day^−1^ of probiotic (enriched with *Bifidobacterium* and *Lactobacillus* 4.8 × 10^10^ colony forming units) or conventional yogurt for 4 weeks played a role in improving the symptoms of constipation during pregnancy [13]. In another study, 500 mL day^−1^ of a probiotic kefir was administered to a group of STC patients for 4 weeks, who showed an increased stool frequency (*p* < 0.001), improved stool consistency (*p*=0.014), and decreased laxative consumption (*p*=0.031) [12]. In contrast, the defecation habits of healthy individuals remained unchanged during the consumption of the yogurt formulation, suggesting that it may not cause any adverse effects in healthy individuals.

### 3.2. Yogurt Effects on Short-Chain Fatty Acids

**Figure 1:**
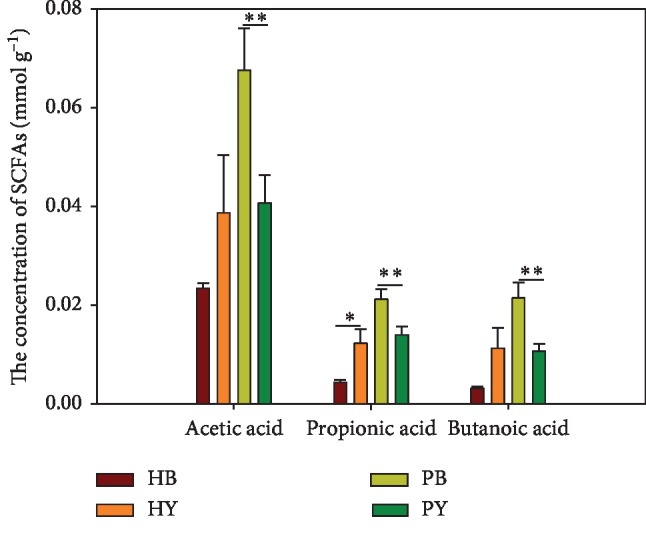
The concentration of short-chain fatty acids in fecal samples from the four groups. HB = healthy individuals before yogurt consumption; HY = healthy individuals after yogurt consumption; PB = patients before yogurt consumption; PY = patients after yogurt consumption.

The concentrations of acetic, propionic, and butanoic acids significantly decreased (*p*=0.014, *p*=0.019, and *p*=0.005) in the fecal samples of patients having ingested the three-strain yogurt formulation for 7 days compared to those observed in patient stool samples prior to consuming the yogurt (Figure 1). However, in the healthy group after yogurt consumption significantly increased propionic acid concentration (*p*=0.04, Figure 1). In our previous study, significant (*p*=0.001) decreases in butanoic acid content were observed in groups of mice that were given three different strains of yogurt. In addition, the acetic and propionic acid contents also showed decreasing trends in the groups of mice receiving the three-strain yogurt formulation compared to those observed in the control group [15]. The use of probiotics to treat constipated patients caused an increase in acetic acid but led to a decreasing trend of propionic and butyric acid, although the use of storyose tetrahydrate significantly increased the propionic acid content, while the acetic and butanoic acid contents also significantly increased after the use of storyose tetrahydrate approximately two weeks [18]. Moreover, in another study, after treating constipated mice with three different types of oligosaccharides, the results showed that all three oligosaccharides increased the proportion of acetic acid and decreased those of propionic and butyric acid in the feces. The increase in the proportion of acetic acid and the decreased concentration of butyric acid in feces should alleviate the symptoms of symptoms constipation [19]. However, the effect of SCFAs on colonic contractility, motility, and transit time remains unclear [20].

### 3.3. Sequence Information and Diversity Index

**Figure 2:**
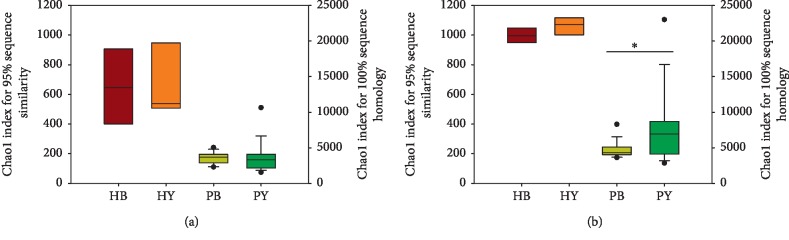
Bacterial similarity and species richness estimates of bacterial 16S rRNA gene sequences obtained by PCR amplification with 95% sequence similarity (a) and 100% sequence homology (b) nucleotide substitutions per nucleotide position. HB = healthy individuals before yogurt consumption; HY = healthy individuals after yogurt consumption; PB = patients before yogurt consumption; PY = patients after yogurt consumption.

A total of 306,165 sequences were obtained by Illumina MiSeq from 40 samples, and the effects of yogurt on the gut bacterial diversity were observed. The estimated OTU numbers were generated to allow 95 and 100% sequence homology of the bacterial 16S rRNA gene sequences using 1600 randomly selected sequences (at the same sequencing depth of all samples). At a 5% sequence dissimilarity level, based on the observed Chao 1 index values, there was no significant differences in fecal microbial diversity between the STC patients before and after they consumed the yogurt (Figure 2(a)). However, at 100% sequence homology level, a significantly higher Chao 1 index value was observed for the STC patients after they consumed yogurt for 7 days (*p*=0.027, Figure 2(b)). From the above results, we can infer that the STC patients who consumed the three-strain yogurt formulation could increase the gut microbial diversity, but the yogurt has little effect on the intestinal microbial diversity in healthy individuals.

**Figure 3:**
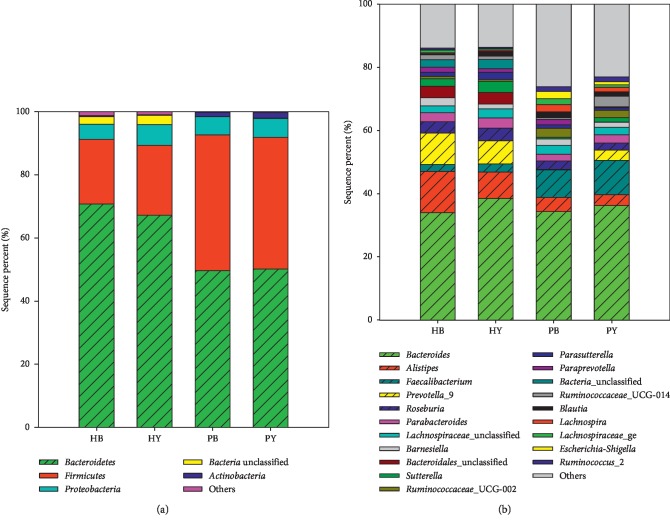
Bacterial community composition at the phylum (a) and genus (b) levels in the fecal samples from the four groups. HB = healthy individuals before yogurt consumption; HY = healthy individuals after yogurt consumption; PB = patients before yogurt consumption; PY = patients after yogurt consumption.

A taxonomic analysis at the phylum level (Figure 3(a)) identified 26 different bacterial phyla in all the samples. The majority of sequences belonged to *Bacteroidetes* (59.82%), *Firmicutes* (31.6%), and *Proteobacteria* (5.68%), which collectively accounted for more than 97% of all the sequences (Figure 3(a)). In the fecal samples obtained from patients before the yogurt treatment, nearly 93% of the sequences belonged to *Bacteroidetes* (49.66 ± 7.79%) and *Firmicutes* (42.99 ± 7.81%), while the proportions of *Proteobacteria* and *Actinobacteria* sequences were lower, 5.75 ± 1.65% and 1.33%, respectively. After consuming the yogurt, the stool samples of the patients also primarily contained sequences of the four abovementioned phyla, and they (50.18, 41.69, 5.99, and 1.73%) did not change significantly (*p* = 0.576, 0.508, 0.844, and 0.878, respectively). Similar results were obtained for the healthy population samples, where the majority of the sequences belonged to the phyla *Bacteroidetes*, *Firmicutes*, and *Proteobacteria* in the stool samples collected both before and after these healthy individuals consumed the yogurt. However, approximately 2.59% of the sequences were unclassified at the phylum level in the healthy population samples.

At the genus level (Figure 3(b)), no significant differences in the percentage of the dominant genera in both the healthy and STC patient groups were observed before and after they consumed the yogurt for 7 days. The top 33 most dominant genera (relative abundance > 0.5%, Table 2) were analyzed. For the STC patient group, 23 genera had relative abundances that changed significantly before and after the patients consumed the yogurt. Among these genera, 7 significantly increased abundances, including *Prevotella*_9, *Bacteroidales*_unclassified, *Sutterella*, *Ruminococcaceae*_UCG-014, *Ruminococcus*_2, *Bifidobacterium*, and *Lachnospiraceae*_NK4A136_group, while those of 16 genera significantly decreased, including *Roseburia*, *Barnesiella*, *Ruminococcaceae*_UCG-002, *Paraprevotella*, *Blautia*, *Lachnospira*, *Lachnospiraceae*_ge, *Escherichia-Shigella*, *Subdoligranulum*, *Ruminococcacea*e_ge, *Ruminococcaceae*_unclassified, *Lachnoclostridium*, *Coprobacter*, *Lachnospiraceae*_UCG-004, *Anaerostipes*, and *Dialister*. Additionally, the relative abundance of genera associated with SCFA production [18], such as *Blatuia*, *Lachnospira*, and *Oscillibacter*, showed decreasing trends. Therefore, we inferred that the decrease in SCFAs in the STC patients after they consumed the yogurt may have been caused by the decrease in the abundances of genera associated with SCFA production. In contrast, the relative abundances of the genera that may promote colonic transit, such as *Faecalibacterium* and *Roseburia* [21], showed an increasing trend in STC patients after they consumed the yogurt.

### 3.4. OTU Difference

**Figure 4:**
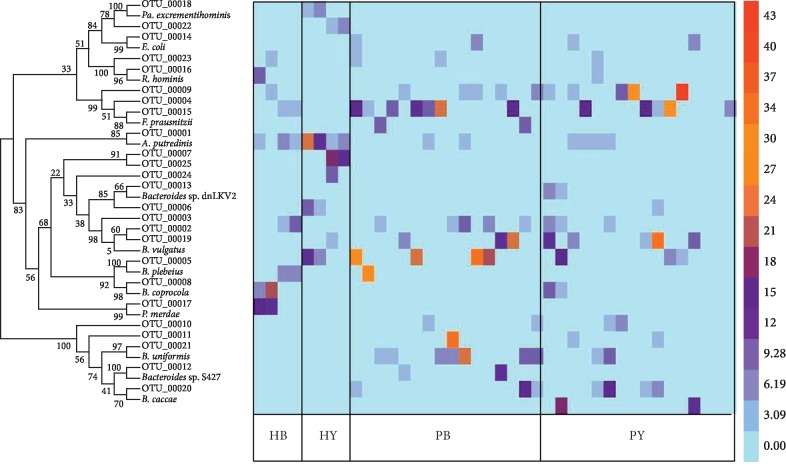
The top 25 most abundant OTUs identified with 100% sequence homology in the samples of the four groups. HB = healthy individuals before yogurt consumption; HY = healthy individuals after yogurt consumption; PB = patients before yogurt consumption; PY = patients after yogurt consumption.

A total of 20,578 OTUs were identified in this study. The names of OTUs were based on the number of sequences. The 25 OTUs with abundances greater than 0.7% were defined as the core OTUs, which represented 40.7% of the total sequences. For the top 25 OTUs based on the BLAST results, there were twelve *Bacteroides* spp., four *Faecalibacterium* spp., one *Alistipes* sp., one *Prevotella* sp., one *Roseburia* sp., one *Parasutterella* sp., one *Parabacteroides* sp., one *Sutterella* sp., one *Escherichia-Shigella* clone, and two uncultured bacterial clones (Figure 4). OTU 00001 was 99% similar to the *Alistipes putredinis* strain ATCC 29800 [22]. Interestingly, the ratio of this bacterium was studied as a novel biomarker of obesity-associated gut dysbiosis in humans [23]. The percentages of OTU 00001 decreased in both the healthy and constipated subjects after they consumed the yogurt for 7 days. OTU 00002 showed 100% identity to the *Bacteroides dorei* isolate HS1_L_3_B_079 [24]. *B. dorei* is a novel strain of *Bacteroides* isolated from the human intestinal tract in 2006 [25]. Interestingly, the abundance of *B. dorei* in fecal samples of children with type 1 diabetes mellitus is significantly higher than that observed in healthy controls, and it can be used as a potential indicator for the detection of type 1 diabetes mellitus [26]. OTUs 00003, 00011, 00019, and 00024 belong to the species *B. vulgatus* and show similarities with different strains [27–30]. *B. vulgatus* is one of the most common microorganisms in the human gastrointestinal tract and has been demonstrated to be a member of the core human intestinal microbiota [26, 31]. Generally, it is believed that *B. vulgatus* is a beneficial symbiotic bacterium in the human intestine, but some studies have shown that intraspecific variation in some strains of *B. vulgatus* can promote or prevent colitis [32–34]. OTUs 00004, 00009, and 00015 showed 99% similarity with different *F. prausnitzii* strains [35–37]*. Faecalibacterium* is the most abundant bacterium in the human intestinal microbiota of healthy adults, representing more than 5% of the total bacterial population [38]. Changes in the abundance of *F. prausnitzii* have been linked to symbiosis in several human disorders [39–41]. OTU 00005 showed 100% identity to *B. plebeius* [42], while both OTUs 00006 and 00021 showed 100% identity to *B. uniformis* strain EBA25-2. The sequences of OTUs 00010, 00011, 00012, 00020, and 00021 were only identified in constipated subjects, and all of these OTUs belonged to *Bacteroides* spp. In contrast, OTUs 00007, 00008, 00022, and 00025 were only identified in healthy subjects and showed 100, 99, 96, and 99% identity to the *Prevotella copri* strain CB7 [43], *B. coprocola* strain M16 [42], *Sutterella wadsworthensis* strain SW7 [44], and uncultured bacterium clone ncd2685f02c1 [45], respectively. *Prevotella* spp. often reside in the mouth and vagina of humans, and these bacteria are associated with respiratory tract anaerobic infections (including aspiration pneumonia, pulmonary abscess, chronic otitis media, and sinusitis) and play a major role in periodontal disease and periodontal abscess [46]. However, the abundance of *P. copri* in fecal samples from children with type 1 diabetes mellitus was significantly higher than that observed in healthy controls [47], and the abundance of *P. copri* may be associated with rheumatoid arthritis [48]. The percentage of OTU 00014 showed significant differences (*p* < 0.05) in the stool samples of STC patients before and after they consumed the yogurt. OTU 00014 showed 100% identity with the *Escherichia coli* strain RM9387 [49]. However, because the use of the 16S rRNA gene alone cannot differentiate *Escherichia* spp. from *Shigella* spp., OTU 00014 belongs to *Escherichia-Shigella.* A similar result was reported after symbiotic treatments for 1 and 3 months, where the abundance of *Escherichia-Shigella* decreased in functional constipation patients [10]. The sequence of OTU 00016 showed 100% identity to the *Roseburia hominis* strain A2-183 [50], one of the most proficient butyrate producers in the human gut [35]. OTU 00017 showed 100% identity to the *Parabacteroides merdae* strain JCM9497 [51], while OTU 00018 showed 100% identity to the *Parasutterella excrementihominis* strain YIT 11859 [52]. Interestingly, a previous study showed that *P. excrementihominis* was associated with irritable bowel syndrome [53].

**Figure 5:**
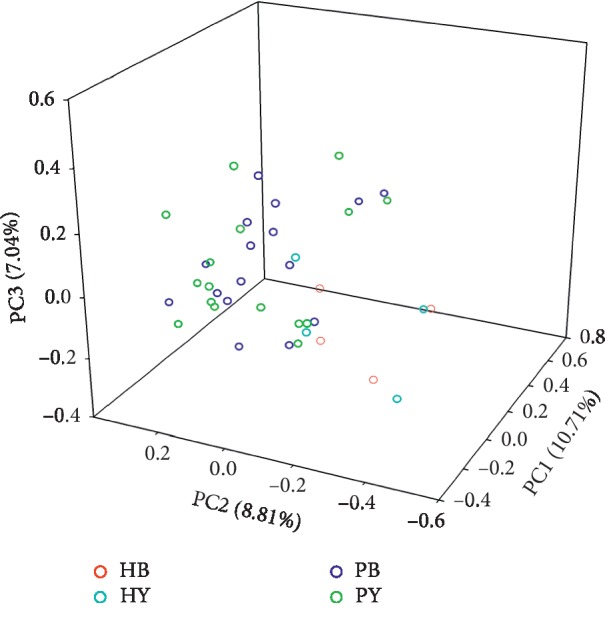
PCA analysis plots based on weighted UniFrac metrics. Each point corresponds to a sample: red circle: HB = healthy individuals before yogurt consumption; cyan circle: HY = healthy individuals after yogurt consumption; blue circle: PB = patients before yogurt consumption; green circle: PY = patients after yogurt consumption.

The diversity of the bacterial composition at the genus level in the stool of individuals before and after consuming the three-strain yogurt formulation was revealed using principal coordinates analysis (PCA) on weighted UniFrac matrixes. Overall, 26.65% of the total variation was explained by the first three principal coordinates (PC1 = 10.71%, PC2 = 8.81%, and PC3 = 7.04%) (Figure 5).

In summary, no differences in the gut microbial diversity were observed in healthy individuals before and after they consumed the yogurt. However, for STC patients, significant differences in the microbial diversity and dominant genera in the stool samples were observed before and after they consumed the three-strain yogurt formulation.

## 4. Conclusion

In this study, the consumption of a three-strain yogurt formulation for 7 days increased the defecation frequency and bacterial diversity in the fecal samples of STC patients but decreased the content of SCFAs in the feces. The results of this study also indicated that yogurt intake could also alter the intestinal bacterial community composition. In short, the results of this study indicate that the intake of the three-strain yogurt formulation promoted intestinal movement and relieved constipation in STC patients.

## Figures and Tables

**Table 1:** The basic physical condition of study people and their clinical response before and after they ate a three-strain yogurt for 7 days.

Subjects	Sex	Age (years)	Weight (kg)	Height (m)	BMI	Defecation frequency (week)	
						Before	After
Healthy	3/1	47.0 ± 10.36	61.5 ± 2.38	1.6 ± 0.07	23.5 ± 1.29	7.0 ± 0	7.0 ± 0
Patient	14/2	46.69 ± 11.45	58.44 ± 9.84	1.63 ± 0.07	21.38 ± 2.53	5.48 ± 2.41	1.81 ± 0.69^*∗∗∗*^

Values are expressed as mean ± SD of three repeats. ^*∗∗∗*^*p* < 0.001 compared with before eating three-strain yogurt.

**Table 2:** Comparisons of healthy people or STC patients before and after they ate a three-strain yogurt for 7 days at the genus level.

Taxon	HB (%)	HY(%)	*p*	PB (%)	PB (%)	*p*
*Bacteroides*	33.0 ± 10.5	36.6 ± 17.8	0.465	30.6 ± 17.0	35.5 ± 21.0	0.317
*Alistipes*	12.2 ± 9.4	8.4 ± 5.0	0.388	4.1 ± 3.4	3.0 ± 2.6	0.252
*Faecalibacterium*	2.4 ± 1.6	2.9 ± 2.4	0.564	9.6 ± 7.6	12.3 ± 12.4	0.129
*Prevotella_9*	10.7 ± 21.4	7.6 ± 15.2	<0.050	0.2 ± 0.9	3.4 ± 14.7	<0.050
*Roseburia*	3.4 ± 5.1	3.6 ± 3.5	0.336	3.1 ± 2.7	2.7 ± 4.7	<0.050
*Parabacteroides*	2.7 ± 0.8	3.1 ± 3.1	0.786	2.4 ± 3.1	2.8 ± 2.8	0.82
*Lachnospiraceae_*unclassified	2.2 ± 1.3	3.0 ± 1.7	0.423	3.2 ± 2.7	2.8 ± 3.2	0.693
*Barnesiella*	3.0 ± 3.7	1.8 ± 1.5	0.371	1.8 ± 2.3	1.4 ± 2.0	<0.050
*Bacteroidales*_unclassified	3.7 ± 0.9	3.8 ± 1.4	0.882	0	0.1 ± 0.1	<0.050
*Sutterella*	2.4 ± 3.3	3.5 ± 5.3	<0.050	0.5 ± 0.8	0.7 ± 1.5	<0.050
*Ruminococcaceae_*UCG-002	0.9 ± 1.0	0.6 ± 0.7	0.642	3.6 ± 7.0	1.7 ± 2.9	<0.050
*Parasutterella*	1.2 ± 1.1	2.0 ± 3.0	0.06	1.4 ± 2.1	0.8 ± 1.2	0.099
*Paraprevotella*	1.5 ± 2.9	0.9 ± 1.8	<0.050	1.7 ± 3.2	0.4 ± 0.7	<0.050
*Ruminococcaceae_*UCG-014	1.7 ± 1.9	1.2 ± 1.4	0.083	0.6 ± 0.8	2.2 ± 3.9	<0.050
*Blautia*	0.3 ± 0.1	1.3 ± 1.9	0.085	2.2 ± 2.6	1.7 ± 1.5	<0.050
*Lachnospira*	0.3 ± 0.4	0.4 ± 0.5	0.766	2.4 ± 2.4	1.3 ± 1.4	<0.050
*Lachnospiraceae_ge*	0.8 ± 0.6	0.6 ± 0.7	0.802	1.7 ± 2.0	0.8 ± 0.5	<0.050
*Escherichia-Shigella*	0.1 ± 0.2	0.04 ± 0.1	0.547	2.0 ± 2.9	1.2 ± 3.1	<0.050
*Ruminococcus_*2	0.6 ± 0.3	0.4 ± 0.3	0.664	1.3 ± 1.3	1.5 ± 2.4	<0.050
*Odoribacter*	1.1 ± 0.2	1.1 ± 0.6	0.156	1.0 ± 1.3	0.7 ± 0.7	0.068
*Subdoligranulum*	0.7 ± 0.7	0.9 ± 0.9	0.058	1.4 ± 1.5	0.7 ± 0.8	<0.050
*Ruminococcaceae_*ge	0.6 ± 0.4	0.5 ± 0.6	0.803	1.4 ± 3.1	1.0 ± 1.8	<0.050
*Ruminococcaceae_*unclassified	0.8 ± 0.6	0.8 ± 0.7	0.201	0.6 ± 1.0	0.2 ± 0.2	<0.050
*Ruminococcus_*1	0.3 ± 0.2	0.7 ± 0.9	0.296	0.9 ± 0.9	0.7 ± 0.8	0.588
*Lachnoclostridium*	0.2 ± 0.2	0.3 ± 0.3	0.551	1.3 ± 1.9	1.0 ± 1.9	<0.050
*Coprobacter*	0	0	—	1.1 ± 2.4	1.0 ± 2.7	<0.050
*Bifidobacterium*	0.2 ± 0.2	0.1 ± 0.1	0.513	0.8 ± 1.0	1.2 ± 3.1	<0.050
*Lachnospiraceae_*UCG-004	0.6 ± 1.1	0.8 ± 1.5	<0.050	0.5 ± 0.9	0.4 ± 0.3	<0.050
*Anaerostipes*	0.1 ± 0.1	0.5 ± 0.6	0.15	0.9 ± 1.3	0.8 ± 1.3	<0.050
*Lachnospiraceae_*NK4A136_group	0.2 ± 0.2	0.1 ± 0.1	0.616	0.7 ± 0.7	1.7 ± 3.1	<0.050
*Dialister*	0.4 ± 0.6	0.2 ± 0.3	0.269	1.0 ± 3.0	0.3 ± 0.5	<0.050

HB = healthy people before eating yogurt; HY = healthy people after eating yogurt; PB = patients before eating yogurt; PY = patients after eating yogurt.

## Data Availability

The PCR product sequencing data generated in this study have been deposited in the Short Read Archive of NCBI under the accession number PRJNA510483, and the other data used to support the findings of this study are available from the corresponding author upon request.

## References

[1] Bharucha A. E., Dorn S. D., Lembo A., Pressman A. (2013). American Gastroenterological Association medical position statement on constipation. *Gastroenterology*.

[2] Koebnick C., Wagner I., Leitzmann P., Stern U., Zunft H. F. (2003). Probiotic beverage Containing *Lactobacillus casei* Shirota improves gastrointestinal symptoms in patients with chronic constipation. *Canadian Journal of Gastroenterology*.

[3] Lembo A., Camilleri M. (2003). Chronic constipation. *New England Journal of Medicine*.

[4] Dinning P. G., Hunt L., Patton V. (2015). Treatment efficacy of sacral nerve stimulation in slow transit constipation: a two-phase, double-blind randomized controlled crossover study. *American Journal of Gastroenterology*.

[5] Bruce Wirta S., Hodgkins P., Joseph A. (2014). Economic burden associated with chronic constipation in Sweden: a retrospective cohort study. *ClinicoEconomics and Outcomes Research*.

[6] Smith B. (1973). Pathologic changes in the colon produced by anthraquinone purgatives. *Diseases of the Colon & Rectum*.

[7] WHO-FAO (2001). *Health and Nutritional Properties of Probiotics in Food Including Powder Milk with Live Lactic Acid Bacteria*.

[8] Singh V. P., Sharma J., Babu S., Rizwanulla, Singla A. (2013). Role of probiotics in health and disease: a review. *Journal of the Pakistan Medical Association*.

[9] Kim S.-E., Choi S. C., Park K. S. (2015). Change of fecal flora and effectiveness of the short-term VSL#3 probiotic treatment in patients with functional constipation. *Journal of Neurogastroenterology and Motility*.

[10] Huang L. S., Kong C., Gao R. Y. (2018). Analysis of fecal microbiota in patients with functional constipation undergoing treatment with synbiotics. *European Journal of Clinical Microbiology & Infectious Diseases*.

[11] Mazlyn M. M., Nagarajah L. H.-L., Fatimah A., Norimah A. K., Goh K.-L. (2013). Effects of a probiotic fermented milk on functional constipation: a randomized, double-blind, placebo-controlled study. *Journal of Gastroenterology and Hepatology*.

[12] Turan I., Dedeli O., Bor S., Ilter T. (2014). Effects of a kefir supplement on symptoms, colonic transit, and bowel satisfaction score in patients with chronic constipation: a pilot study. *The Turkish Journal of Gastroenterology*.

[13] Mirghafourvand M., Rad A. H., Alizadeh S. M. C., Fardiazar Z., Shokri K. (2016). The effect of probiotic yogurt on constipation in pregnant women: a randomized controlled clinical trial. *Iranian Red Crescent Medical Journal*.

[14] Kim B.-K., Choi I. S., Kim J., Han S. H., Suh H. J., Hwang J.-K. (2017). Effects of fermented milk with mixed strains as a probiotic on the inhibition of loperamide-induced constipation. *Korean Journal for Food Science of Animal Resources*.

[15] Liu C.-J., Tang X.-D., Yu J., Zhang H.-Y., Li X.-R. (2017). Gut microbiota alterations from different Lactobacillus probiotic-fermented yoghurt treatments in slow-transit constipation. *Journal of Functional Foods*.

[16] Thompson J. R., Marcelino L. A., Polz M. F. (2002). Heteroduplexes in mixed-template amplifications: formation, consequence and elimination by “reconditioning PCR”. *Nucleic Acids Research*.

[17] Schloss P. D., Westcott S. L., Ryabin T. (2009). Introducing mothur: open-source, platform-independent, community-supported software for describing and comparing microbial communities. *Applied and Environmental Microbiology*.

[18] Zang K., Jiang Y., Sun Y. (2018). Relationship between microecologics and the expression of short chain fatty acids synthesis genes in key bacterial genera in the regulation of intestinal flora structure in populations with constipation and diarrhea. *Food Science*.

[19] Wang L., Hu L., Yan S. (2017). Effects of different oligosaccharides at various dosages on the composition of gut microbiota and short-chain fatty acids in mice with constipation. *Food & Function*.

[20] Camilleri M. (2013). Peripheral mechanisms in irritable bowel syndrome reply. *New England Journal of Medicine*.

[21] Parthasarathy G., Chen J., Chen X. (2016). Relationship between microbiota of the colonic mucosa vs feces and symptoms, colonic transit, and methane production in female patients with chronic constipation. *Gastroenterology*.

[22] Paster B. J., Dewhirst F. E., Olsen I., Fraser G. J. (1994). Phylogeny of *Bacteroides*, Prevotella, and *Porphyromonas* spp. and related bacteria. *Journal of Bacteriology*.

[23] Sabater-Masdeu M., Palomo-Buitrago M. E., Comas F. (2018). The ratio of Erysipelotrichaceae/Alistipes putredinis as a novel biomarker of obesity-associated gut dysbiosis in humans. *European Journal of Clinical Investigation*.

[24] Leonard M. T., Davis-Richardson A. G., Ardissone A. N. (2014). The methylome of the gut microbiome: disparate Dam methylation patterns in intestinal *Bacteroides dorei*. *Frontiers in Microbiology*.

[25] Bakir M. A., Sakamoto M., Kitahara M., Matsumoto M., Benno Y. (2006). Bacteroides dorei sp. nov., isolated from human faeces. *International Journal of Systematic and Evolutionary Microbiology*.

[26] Qin J., Li R., Raes J. (2010). A human gut microbial gene catalogue established by metagenomic sequencing. *Nature*.

[27] Xu J., Mahowald M. A., Ley R. E. (2007). Evolution of symbiotic bacteria in the distal human intestine. *PLoS Biology*.

[28] Sakamoto M., Suzuki N., Benno Y. (2010). hsp60 and 16S rRNA gene sequence relationships among species of the genus Bacteroides with the finding that Bacteroides suis and Bacteroides tectus are heterotypic synonyms of Bacteroides pyogenes. *International Journal of Systematic and Evolutionary Microbiology*.

[29] Shinohara K., Ohashi Y., Kawasumi K., Terada A., Fujisawa T. (2010). Effect of apple intake on fecal microbiota and metabolites in humans. *Anaerobe*.

[30] Lagkouvardos I., Pukall R., Abt B. (2016). The Mouse Intestinal Bacterial Collection (miBC) provides host-specific insight into cultured diversity and functional potential of the gut microbiota. *Nature Microbiology*.

[31] Tap J., Mondot S., Levenez F. (2009). Towards the human intestinal microbiota phylogenetic core. *Environmental Microbiology*.

[32] Rath H. C., Wilson K. H., Sartor R. B. (1999). Differential induction of colitis and gastritis in HLA-B27 transgenic rats selectively colonized with Bacteroides vulgatus or *Escherichia coli*. *Infection and Immunity*.

[33] Waidmann M., Bechtold O., Frick J.-s. (2003). Bacteroides vulgatus protects against Escherichia coli-induced colitis in gnotobiotic interleukin-2-deficient mice. *Gastroenterology*.

[34] Frick J. S., Fink K., Kahl F., Niemiec M. J., Quitadamo M., Autenrieth I. B. K. (2007). Identification of commensal bacterial strains that modulate Yersinia enterocolitica and dextran sodium sulfate-induced inflammatory responses: implications for the development of probiotics. *Infection and Immunity*.

[35] Barcenilla A., Pryde S. E., Martin J. C. (2000). Phylogenetic relationships of butyrate-producing bacteria from the human gut. *Applied and Environmental Microbiology*.

[36] Li M., Wang B., Zhang M. (2008). Symbiotic gut microbes modulate human metabolic phenotypes. *Proceedings of the National Academy of Sciences*.

[37] Blachier F., Foditsch C., Santos T. M. A. (2014). Isolation and characterization of Faecalibacterium prausnitzii from calves and piglets. *PLoS One*.

[38] Miquel S., Martín R., Rossi O. (2013). Faecalibacterium prausnitzii and human intestinal health. *Current Opinion in Microbiology*.

[39] Sokol H., Pigneur B., Watterlot L. (2008). Faecalibacterium prausnitzii is an anti-inflammatory commensal bacterium identified by gut microbiota analysis of Crohn disease patients. *Proceedings of the National Academy of Sciences of the United States of America*.

[40] Machiels K., Joossens M., Sabino J. (2014). A decrease of the butyrate-producing species *Roseburia hominis* and *Faecalibacterium prausnitzii* defines dysbiosis in patients with ulcerative colitis. *Gut*.

[41] Jiang H., Ling Z., Zhang Y. (2015). Altered fecal microbiota composition in patients with major depressive disorder. *Brain, Behavior, and Immunity*.

[42] Kitahara M., Sakamoto M., Ike M., Sakata S., Benno Y. (2005). Bacteroides plebeius sp. nov. and Bacteroides coprocola sp. nov., isolated from human faeces. *International Journal of Systematic and Evolutionary Microbiology*.

[43] Hayashi H., Shibata K., Sakamoto M., Tomita S., Benno Y. (2007). Prevotella copri sp. nov. and Prevotella stercorea sp. nov., isolated from human faeces. *International Journal of Systematic and Evolutionary Microbiology*.

[44] Mukhopadhya I., Hansen R., Nicholl C. E. (2011). A comprehensive evaluation of colonic mucosal isolates of Sutterella wadsworthensis from inflammatory bowel disease. *PLoS One*.

[45] Kong H. H., Oh J., Deming C. (2012). Temporal shifts in the skin microbiome associated with disease flares and treatment in children with atopic dermatitis. *Genome Research*.

[46] Tanaka S., Yoshida M., Murakami Y. (2008). The relationship of *Prevotella intermedia*, *Prevotella nigrescens* and *Prevotella melaninogenica* in the supragingival plaque of children, caries and oral malodor. *Journal of Clinical Pediatric Dentistry*.

[47] Davis-Richardson A. G., Ardissone A. N., Dias R. (2014). Bacteroides dorei dominates gut microbiome prior to autoimnnunity in Finnish children at high risk for type 1 diabetes. *Frontiers in Microbiology*.

[48] Scher J. U., Sczesnak A., Longman R. S. (2013). Expansion of intestinal Prevotella copri correlates with enhanced susceptibility to arthritis. *Elife*.

[49] Yan X., Fratamico P. M., Bono J. L., Baranzoni G. M., Chen C.-Y. (2015). Genome sequencing and comparative genomics provides insights on the evolutionary dynamics and pathogenic potential of different H-serotypes of Shiga toxin-producing *Escherichia coli* O104. *BMC Microbiology*.

[50] Travis A. J., Kelly D., Flint H. J., Aminov R. I. (2015). Complete genome sequence of the human gut symbiont Roseburia hominis. *Genome Announcements*.

[51] Sakamoto M., Benno Y. (2006). Reclassification of Bacteroides distasonis, Bacteroides goldsteinii and Bacteroides merdae as Parabacteroides distasonis gen. nov., comb. nov., Parabacteroides goldsteinii comb. nov. and Parabacteroides merdae comb. nov. *International Journal of Systematic and Evolutionary Microbiology*.

[52] Nagai F., Morotomi M., Sakon H., Tanaka R. (2009). Parasutterella excrementihominis gen. nov., sp. nov., a member of the family Alcaligenaceae isolated from human faeces. *International Journal of Systematic and Evolutionary Microbiology*.

[53] Chen Y.-J., Wu H., Wu S.-D. (2018). Parasutterella, in association with irritable bowel syndrome and intestinal chronic inflammation. *Journal of Gastroenterology and Hepatology*.

